# Synthesis of New GABA_A_ Receptor Modulator with Pyrazolo[1,5-a]quinazoline (PQ) Scaffold

**DOI:** 10.3390/ijms20061438

**Published:** 2019-03-21

**Authors:** Gabriella Guerrini, Claudia Vergelli, Niccolò Cantini, Maria Paola Giovannoni, Simona Daniele, Maria Paola Mascia, Claudia Martini, Letizia Crocetti

**Affiliations:** 1 Dipartimento Neurofarba, sezione Farmaceutica e Nutraceutica, Università di Firenze, Via Ugo Schiff 6, Sesto Fiorentino, 50019 Firenze, Italy; claudia.vergelli@unifi.it (C.V.); niccolo.cantini@unifi.it (N.C.); mariapaola.giovannoni@unifi.it (M.P.G.); letizia.crocetti@unifi.it (L.C.); 2 Dipartimento di Farmacia, Università di Pisa, Via Bonanno 6, 56126 Pisa, Italy; simona.daniele@unipi.it (S.D.); claudia.martini@unipi.it (C.M.); 3 CNR—Institute of Neuroscience, Cagliari, Cittadella Universitaria, 09042 Monserrato, Italy; mariapaola.mascia@cnr.it

**Keywords:** GABA_A_ receptor, high affinity benzodiazepine site, low affinity benzodiazepine site, pyrazolo[1,5-a]quinazoline, organic synthesis, electrophysiological studies, *Xenopus* oocytes

## Abstract

We previously published a series of 8-methoxypirazolo[1,5-a]quinazolines (PQs) and their 4,5-dihydro derivatives (4,5(*H*)PQ) bearing the (hetero)arylalkylester group at position 3 as ligands at the γ-aminobutyric type A (GABA_A_) subtype receptor. Continuing the study in this field, we report here the design and synthesis of 3-(hetero)arylpyrazolo[1,5-a]quinazoline and 3-(hetero)aroylpyrazolo[1,5-a]quinazoline 8-methoxy substituted as interesting analogs of the above (hetero)arylalkylester, in which the shortening or the removal of the linker between the 3-(hetero)aryl ring and the PQ was performed. Only compounds that are able to inhibit radioligand binding by more than 80% at 10 μM have been selected for electrophysiological studies on recombinant α1β2γ2L GABA_A_ receptors. Some compounds show a promising profile. For example, compounds **6a** and **6b** are able to modulate the GABA_A_R in an opposite manner, since **6b** enhances and **6a** reduces the variation of the chlorine current, suggesting that they act as a partial agonist and an inverse partial agonist, respectively. The most potent derivative was 3-(4-methoxyphenylcarbonyl)-8-methoxy-4,5-dihydropyrazolo[1,5-a] quinazoline **11d**, which reaches a maximal activity at 1 μM (+54%), and it enhances the chlorine current at ≥0.01 μM. Finally, compound **6g**, acting as a null modulator at α1β2γ2L, shows the ability to antagonize the full agonist diazepam and the potentiation of CGS 9895 on the new α+/β− ‘non-traditional’ benzodiazepine site.

## 1. Introduction

γ-aminobutyric type A (GABA_A_) receptors Type A (GABA_A_R) and Type B (GABA_B_R) are the principal inhibitory neurotransmitter receptors in the mammalian brain, and are the targets of many important drugs on the market for a variety of neurological conditions, including epilepsy, anxiety, spasticity, pain, and psychiatric illness [1]. The neurotransmitter GABA produces slow (sub-second) synaptic response by interaction with the GABA_B_Rs, belonging to the G-protein-coupled receptors (GPCR) category, or exerts fast (<10 ms) and powerful synaptic inhibition by acting on the GABA_A_Rs, which is a Cys-loop pentameric ligand gated ion channel (LGIC). This last family of receptors also includes the nicotinic acetylcholine receptors (nAChRs), the 5-hydroxytriptamine type 3 receptors (5-HT_3_Rs), and the glycine receptors, all of which show a similar topology, namely five homologous subunits assembled to form a central ion-conducting pore. Each subunit is formed by an N-terminal extracellular domain (ECD), four α-helices transmembrane domains (TM1–TM4), in which the TM2 of all the subunits is arranged to form the inner pore, and a C-terminal intracellular domain (ICD). To date, for the human GABA_A_R, 19 genes encoding the protein receptor have been sequenced and classified as different families, based on the degree of homology: α1–6, β1–3, γ1–3, δ, ε, π, θ, and ρ1–3 [2]. Although the combination of all the subunits could contribute to form the pentamer, α and β subunits are necessary to obtain a fully functional receptor. Subunit γ contributes to form the most abundant GABA_A_ receptor subtype in the central nervous system (CNS), which is composed by 2α2β1γ subunits. Depending on the subunit isoforms (α1–6, β1–3, γ1–3) and regional brain distribution, each subtype receptor (α1βγ2, α2βγ2, α3βγ2, α4βγ2, α5βγ2, and α5βγ2) modulates different brain functions, and in general, its expression at synaptic or extrasynaptic sites mediates phasic or tonic inhibition [3,4]. The δ subunit can co-exist with αβ subunits to form a functional pentamer located exclusively extrasynaptically, or form the GABA_A_R subtype that spontaneously opens the gate, even in the absence of a neurotransmitter [5,6]. The principal binding sites of GABA_A_R are sited in the extracellular domain between two adjacent subunits; the first one is known as the “principal” (conventionally indicated with plus or +), and the other one is called the “complementary” subunit (indicated with minus or −) [4]. Thus, the two orthosteric GABA binding sites are located at the α+/β− interfaces, while the “benzodiazepine site” is located at the α+/β− interface. Recently, a renewed search for GABA_A_R has identified a new interaction site, which is similar to ‘benzodiazepine site’ and located at the homologous interface (α+/β−) [7,8,9]. The α subunit of this site contains the same protein loop sequences of the principal ‘benzodiazepine site α’ and, analogously to this latter, the six α isoforms and the three β isoforms in concert contribute to form the binding site [9]. This α+/β− ‘non-traditional’ benzodiazepine site was first described as the site for diazepam, which is able to produce a less specific GABA potentiation at micromolar concentration. The binding of diazepam at α+/β− is independent of the γ subunit, and it is insensible to flumazenil [10]. The first ligand discovered for this new site [11,12] was CGS 9895 (2-p-methoxyphenylpyrazolo[4,3-c]quinolin-3-(5H)-one), which is already known as a high affinity benzodiazepine site (α+/β−) antagonist. CGS 9895 showed also the ability to enhance the GABA currents at the α+/β− site, working as an agonist. Moreover, the binding to the low-affinity α+/β− binding site is not strictly bound to antagonistic activity at the benzodiazepine site. In fact, in a previous work, it has been demonstrated that also lorazepam and the imidazopyridine zolpidem in GABA_A_ receptors devoid of the γ subunit were able to antagonize the positive modulatory effect of ligands chemically related to rimonabant [13]. Further studies afforded the identification of a number of selective/high affinity α+/β− site ligands [14,15]. In fact, as suggested in the paper of Varagic et al. [14], the GABA_A_ receptor allosteric modulation by interaction with the new α+/β− site, as exerted by analogues of CGS 9895, is not dependent on the GABA_A_ benzodiazepine-binding site (α+/β−). In fact, the authors demonstrated that subtype receptors α6β3 and α6β3γ2 containing the same alpha subunit were modulated by some pyrazoloquinolines and pyrazolopyrimidinones, which did not interact with the ‘benzodiazepine site’, since the interface α+/β− containing the α6 forms the well-known ‘diazepam-insensitive’ receptors. Even Knutson et al. [15] reported the study of new α6β3 not cytotoxic ligands as potential candidates for the treatment of CNS disorder and useful tools for drug development.

The achievement of the first crystal structure of the β3 homopentamer human GABA_A_ receptor at 3Å resolution [16] strongly improved the knowledge of the structural features of GABA_A_R. These results were recently corroborated by Zhu et al. [17], who obtained two high-resolution structures of GABA_A_ receptor, in complex with GABA and flumazenil, using the cryo-electron microscopy. This study also revealed the presence of two sites located at equivalent positions to the GABA and benzodiazepine sites at the γ/β and α/β interfaces (this last one has already been identified and is known as the α+/β− ‘non-traditional benzodiazepine’ site) as potential targets for new drugs.

**Figure 1:**
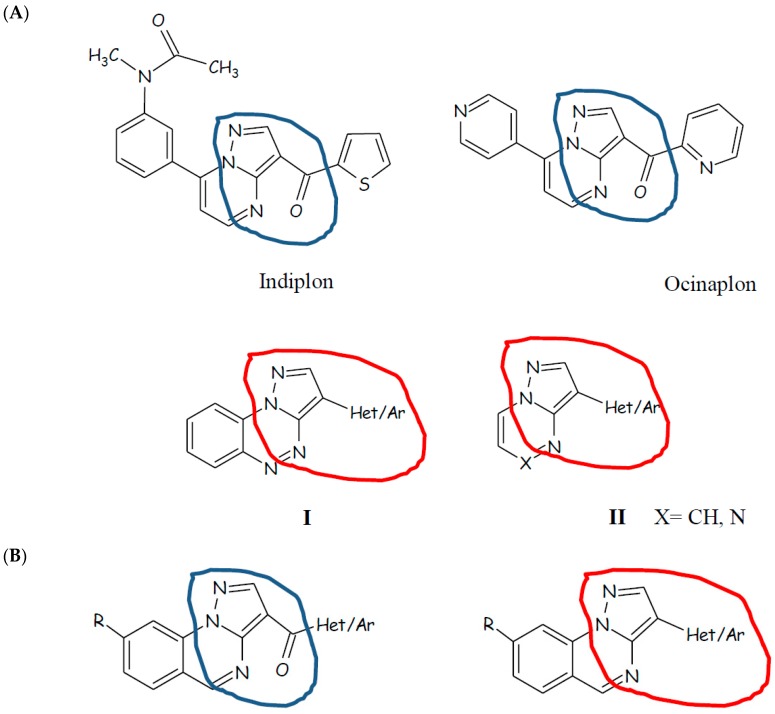
Panel (**A**): γ-aminobutyric type A (GABA_A_) receptor subtype ligands with chemical structures strictly related to the new pyrazoloquinazoline (PQ). Panel (**B**): general structure of new synthesized PQ compounds. Blue circle 3-aroylpyrazolopyrimidine moiety; Red circle 3-arylpyrazolopyrimidina moiety.

Our research group in a previous work reported the design and synthesis of new 8-methoxypyrazolo[1,5-a]quinazoline (PQ) and their 4,5-dihydro derivatives (4,5(*H*)PQ) bearing the (hetero)arylalkylester (3-Het/ArCH_2_COO–) group at position 3. These ligands showed high binding affinity (K_i_ = 0.2–34 nM) and anxiolityc-like/antihyperalgesic activity [18]. Continuing the study on ligands at the GABA_A_ subtype receptor, we considered the shortening or the removal of the linker between the 3-(hetero)aryl ring and the PQ core as an interesting chemical modification of the previously reported ester derivatives. Thus, the synthesis of 8-methoxy 3-(hetero)arylpyrazolo[1,5-a]quinazoline and 3-(hetero)aroylpyrazolo[1,5-a]quinazoline was planned. These compounds are most likely more metabolically stable then ester derivatives [18], and are structurally strictly related to compounds that have been claimed to be GABA_A_ subtype receptor ligands such as Indiplon, Ocinaplon [4,19,20], and compounds of type **I** and **II** [21,22,23] (Figure 1, Panel A). All of these compounds belong to the pyrazolopyrimidines, triazolophtalazine, or pyrazolobenzotriazine family, whose core is easily recovered in our new synthesized PQ compounds (Figure 1, Panel B).

All new compounds were evaluated for their ability to displace [3H]flumazenil(Ro-151788) from its specific binding to Bz receptors in bovine membrane samples. Moreover, chlorine current on recombinant GABA_A_ receptors of the α1β2γ2L type (expressed in frog oocytes of the *Xenopus laevis* species) was limited to those compounds that are able to inhibit radioligand binding by more than 80% at 10 μM. Additionally, for the most interesting compounds, the modulation on the new α+/β− ‘non-traditional’ benzodiazepine site was evaluated.

## 2. Results and Discussion

### 2.1. Chemistry

**Scheme 1:**
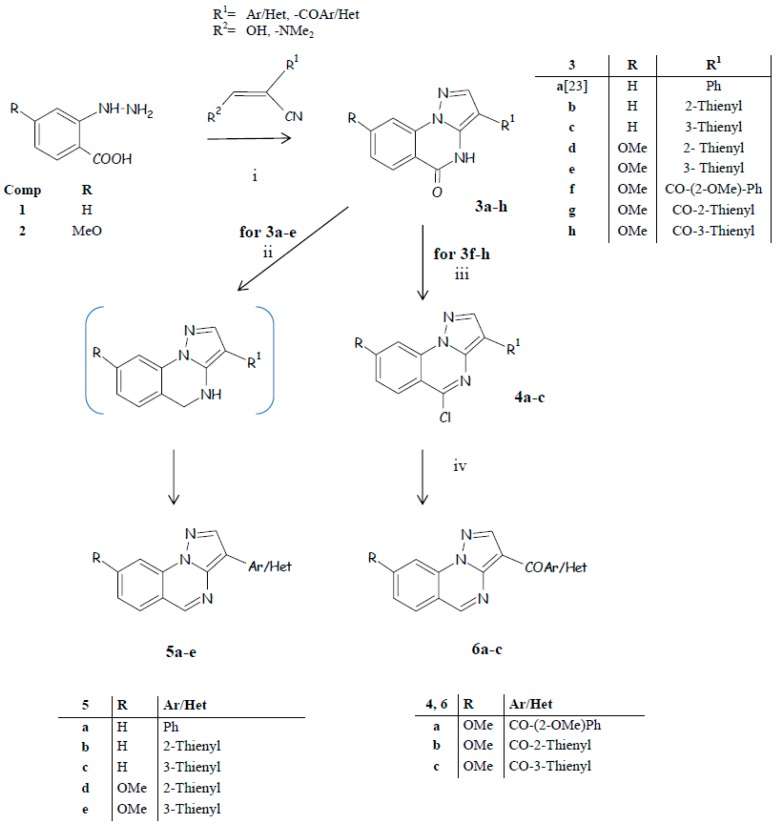
Synthesis of pyrazolo[1,5-a]quinazolin-4(5H)-ones and pyrazolo[1,5-a]quinazolines bearing an aryl/heteroaryl ring or aroyl/heteroaroyl group at position 3. Reagents and conditions: (i) AcOH or dimethylformamide (DMF)/AcONa; (ii) anhydrous THF and LiAlH_4_/THF then air; (iii) POCl_3_/PCl_5_,N,N-diisopropylethylamine (DIPEA); (iv) EtOH, HCOONH_4_, Pd/C 10%.

**Scheme 2:**
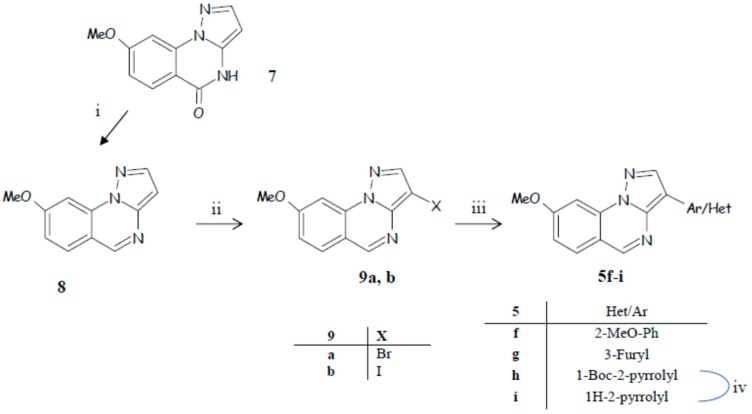
Synthesis of 3-(hetero)arylpyrazolo[1,5-a]quinazoline. Reagents and conditions: (i) anhydrous THF and LiAlH_4_/THF; then, toluene, 10% Pd/C. (ii) Br_2_ or NIS, CH_2_Cl_2_; (iii) Suzuki conditions, arylboronic acids, ethanol abs., (PPh_3_)_4_Pd tetrakis(triphenylphosphine)palladium (0), sodium carbonate 2 M; (iv) THF/MeONa.

**Scheme 3:**
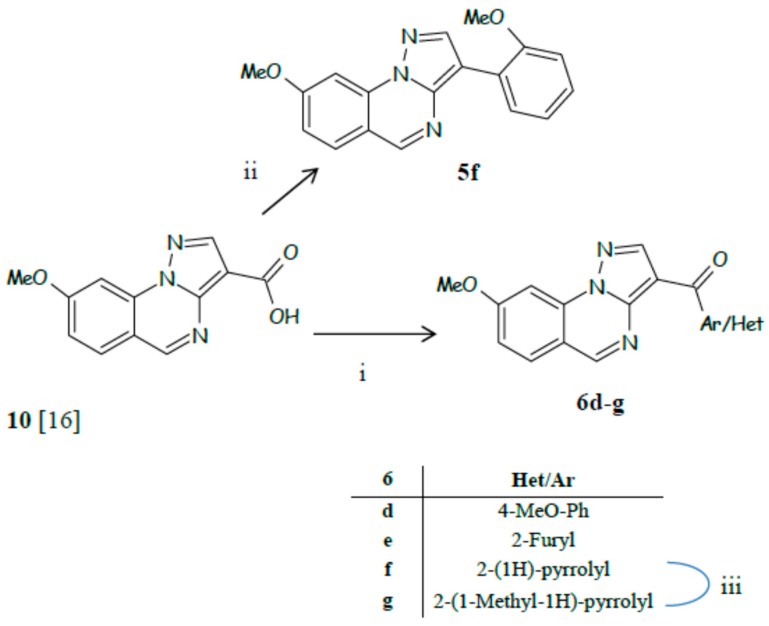
Synthesis of 3-(hetero)aroylpyrazolo[1,5-a]quinazoline derivatives. Reagents and conditions: (i) For compound **6d** Eaton’s reagent and anisole; for **6e**–**g**: SOCl_2_ or Cl_3_CCN/PPh_3_, then SnCl_4_ and suitable ArH/HetH; (ii) SOCl_2_, 2-MeO-PhB(OH)_2_, toluene, Na_2_CO_3_ sol. 2 M; (iii) DMF, K_2_CO_3_, MeI.

Two synthetic approach were developed to obtain the desired 3-(hetero)arylpyrazolo[1,5-a]quinazolines or 3-(hetero)aroylpyrazolo[1,5-a]quinazolines:

1- condensation and next cyclization of the hydrazinobenzoic acid with suitable propanenitrile to directly obtain the pyrazolo[1,5-a]quinazoline scaffold bearing at position 3 the proper (hetero)aryl or (hetero)aroyl group (Scheme 1);
2- introduction of the (hetero)aryl or (hetero)aroyl group through a functionalization of the position 3 (e.g., –H, –COOH) working on the tricyclic system that was previously synthesized (Scheme 2 and Scheme 3).

In the first approach (Scheme 1), the 2-hydrazinobenzoic acid or the 4-methoxy-2-hydrazinobenzoic acid **1** and **2**, [18] were reacted in AcOH or DMF/AcONa, with the appropriate 2-(hetero)aryl-3-oxapropanenitrile [21] or the corresponding 2-(hetero)aroyl-3-(dimethylamino)acrylonitrile [24], to obtain the final 3-(hetero)aryl (**3a [25]**, **3b**–**e**) and 3(heteroaroyl)pyrazolo[1,5-a]quinazolin-4(5*H*)-ones (**3f**–**h**), respectively.

The aromatization of the quinazoline ring was obtained for compounds **3a**–**e** by treatment with LiAlH_4_ in anhydrous THF, and further oxidation in the same vessel reaction (the corresponding 4,5-dihydropyrazolo[1,5-a]quinazolines were not isolated) to directly give the aromatic compounds **5a**–**e**. Instead, compounds **3f**–**h** were transformed into the corresponding 5-chloroderivatives (type **4** compounds), and the next reduction with HCOONH_4_ and 10% Pd/C yielded the final desired compounds **6a**–**c**, without showing the corresponding dihydroderivatives.

In the second approach, modifications of position 3 of the pyrazolo[1,5-a]quinazoline scaffold were performed, depending on the final designed product (Scheme 2 and Scheme 3). For the introduction of the (hetero)aryl ring, the Suzuki coupling reaction was carried out (Scheme 2). The key intermediate **8** was obtained from **7**, the 8-methoxypyrazolo[1,5-a]quinazolin-5(4H)-one, by treatment with LiAlH_4_/THF, and the next dehydrogenation with 10% Pd/C and toluene; compound **7**, in turn, was achieved by decarboxylation of ethyl 8-methoxy-5-oxo-4,5-dihydropyrazolo[1,5-a] quinazoline 3-carboxylate [18]. Halogenation of position 3 of the PQ core, by bromine or N-iodosuccinimide (NIS), gave **9a**,**b**, which were used as counterparts with the suitable boronic acid (2-MeO-phenyl-, 3-furan-, and 1-Boc-2-pyrrolboronic acid) in the Suzuki coupling reaction, yielding compounds **5f**–**h**. The best yields were obtained starting from compound **9b**. The deprotection of 3-(1-Boc-2-pyrrolyl)-8-methoxypyrazolo[1,5-a]quinazoline, **5h**, by THF/MeONa, gave compound **5i**.

For the introduction of a keto group at position 3, still working on the tricyclic system, the 8-methoxypyazolo[1,5-a]quinazoline-3-carboxylic acid **10 [18]** was used as the starting material. The 3-(4-methoxyphenylcarbonyl)-8-methoxypyrazolo[1,5-a]quinazoline **6d** was obtained with Eaton’s reagent and anisole, while the other compounds were obtained through the 3-acylchloride intermediate. The attempt to obtain the 3-carbonylderivatives by Suzuki coupling reaction failed, since for example, when using 2-MeO-phenylboronic acid as the counterpart, unexpectedly, only compound **5f** was recovered, evidencing CO elimination and Ar–Ar coupling (Scheme 3). Thus, the Friedel–Craft reaction was applied and, after treatment of the 8-methoxypyazolo[1,5-a]quinazoline-3-carboxylic acid **9** with SOCl_2_ or Cl_3_CCN and PPh_3_ to give the 3-acylchloride intermediate, the appropriate (hetero)aryl was added, and the final products **6e**–**g** were obtained. Compound **6g** was also obtained by alkylation of **6f** with DMF, K_2_CO_3_, and methyl iodide, but in low yield.

**Scheme 4:**
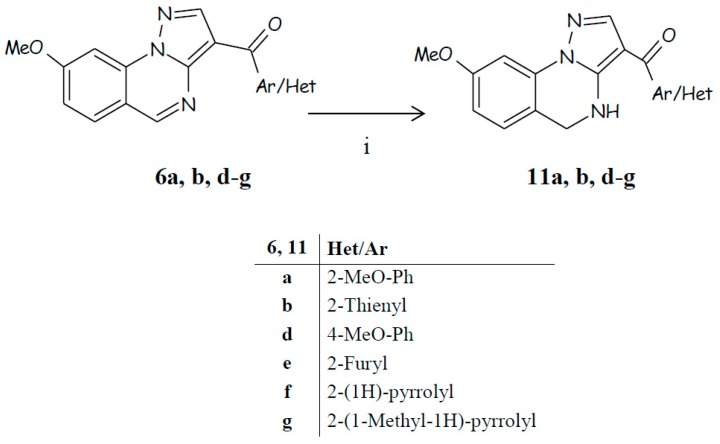
Synthesis of 3-(hetero)aroyl-4,5-dihydropyrazolo[1,5-a]quinazolines. Reagents and conditions: (i) AcOH/NaBH_3_CN.

Finally, type **6** compounds were transformed into the corresponding 4,5-dihydroderivatives by treatment with NaBH_3_CN in AcOH, obtaining compounds **11a**, **b**, and **d**–**g**, as shown in Scheme 4. When 3-(hetero)aryl substituted compounds (type **5**) were reacted in the same conditions, the corresponding 4,5-dihydroderivatives were evidenced only in the reaction mixture by TLC, and were not isolated as pure compounds, because after the normal work up, they were converted again into the starting material.

### 2.2. Biological Evaluation

All of the new compounds were previously evaluated for their ability to displace [^3^H]flumazenil (Ro-151788) from its specific binding to Bz receptors in bovine membrane samples. Only compounds that were able to inhibit radioligand binding by more than 80% at 10 μM (**5d**–**f**, **6a**–**d**, **6f**,**g**, **11a**,**b**,**d**) were further selected for electrophysiological studies on recombinant α1β2γ2L GABA_A_ receptors.

**Figure 2:**
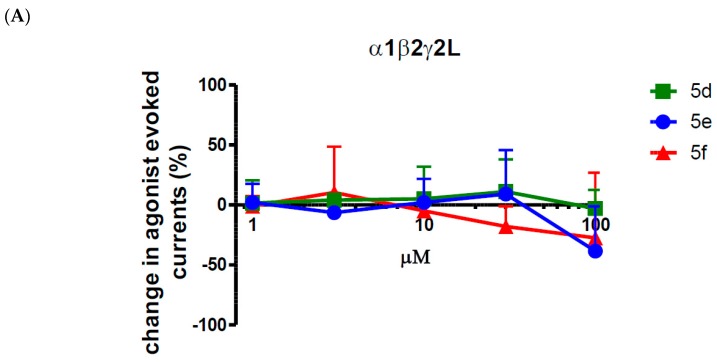

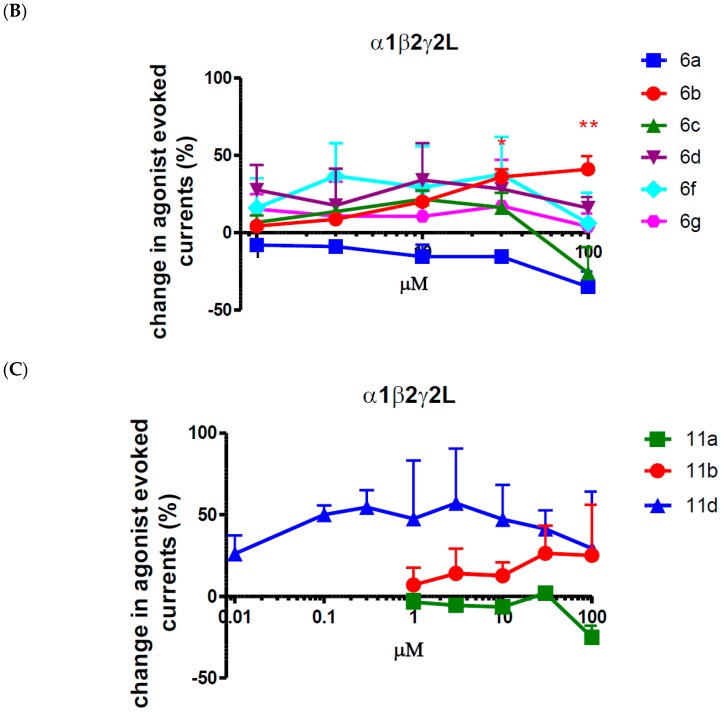
Concentration-response curves of compounds (**A**) **5d**–**f**, (**B**) **6a**–**d**, **6f**,**g**, and (**C**) **11a**,**b**,**d** on GABA-induced Cl^−^ currents in *Xenopus* oocytes expressing recombinant α1β2γ2L GABA_A_ receptors. Data are expressed as the percentage modulation of the response induced by GABA at EC_5–10_ values, and are the mean ± S.E.M of values obtained from two to nine oocytes. ** *p* < 0.01; * *p* < 0.05 Kruskal–Wallis test followed by the Dunn post-hoc test.

Recombinant α1β2γ2L GABA_A_ receptors were expressed in *Xenopus laevis* oocytes, and the effects of compounds tested at 1 to 100 μM were assessed on the modulation of GABA_A_ receptor function (Figure 2). The three sections of Figure 2 respectively report the experiments of pyrazoloquinazoline 3-(hetero)aryl substituted **5d**–**f** (section a), 3-(hetero)aroyl substituted **6a**–**d**, **6f**,**g** (section b), and 4,5-dihydropyrazoloquinazoline-3-(hetero)aroyl substituted **11a**,**b**,**d** (section c).

As evident in Figure 2 (section A), the first group of compounds (**5d**–**f**) was not able to modulate the GABA_A_ function through acting as a null modulator or antagonist. Among compounds **6a**–**d**,**f**,**g** bearing an aroyl moiety at position 3 (Figure 2, section B), the 3-(2-methoxyphenylcarbonyl) -8-methoxypyrazolo[1,5-a]quinazoline **6a** slightly, but not significantly, inhibited the GABA_A_ receptor function at 100 μM. On the contrary, the 3-(thien-2-yl-carbonyl)-8-methoxypyrazolo[1,5-a]quinazoline **6b**, slightly but significantly enhanced the GABA_A_ receptor function until 100 μM, showing an EC_50_ = 11.97 μM and acting as a partial agonist (E_max_ + 42% at 100)

For the 4,5-dihydroderivatives **11a**,**b**,**d**, which are reported in the third part of Figure 2 (section C), compound **11d** stands out as enhancing the GABA_A_ receptor function and reaching a maximum of activity at 1 μM (Emax + 54%), but maintaining a slightly positive variation in chloride current even at ≥0.01 μM.

**Figure 3:**
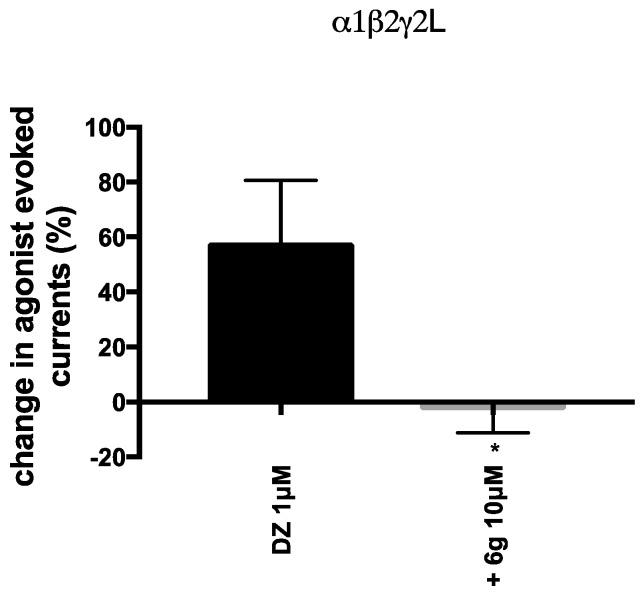
Compound **6g** antagonized the potentiation of GABA-induced Cl^−^ currents by diazepam in *Xenopus* oocytes expressing recombinant α1β2γ2L GABA_A_ receptors. Data are expressed as the percentage potentiation of the response induced by GABA at EC_5–10_ values, and are the mean ± SEM of values obtained from four oocytes. *p* < 0.05 Mann–Whitney test.

In order to confirm whether those compounds that are null modulators act at the benzodiazepine binding site, **6g** as a representative sample was evaluated for its ability to antagonize the full agonist diazepam. Figure 3 clearly demonstrated that the null modulator **6g** is able to completely abolish the potentiation of the GABA_A_ receptor function induced by 1 μ diazepam at 10 μM.

**Figure 4:**
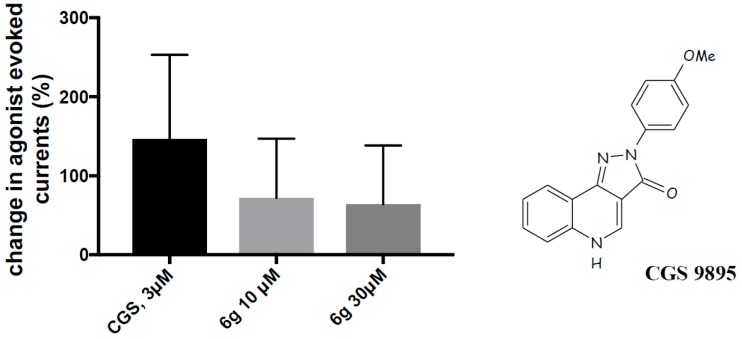
Compound **6g** reduced the potentiation of GABA-induced Cl^−^ currents by CGS 9895 (2-p-methoxyphenylpyrazolo[4,3-c]quinolin-3-(5H)-one) in *Xenopus* oocytes expressing recombinant α1β2 GABA_A_ receptors. Data are expressed as the percentage potentiation of the response induced by GABA at EC_5–10_ values, and are the mean ± SEM of values obtained from three to six oocytes.

Recently, other low-affinity sites for benzodiazepine, in addition to the high-affinity benzodiazepine binding site, have been discovered [9,17,26]. Among them, the site located in the extracellular domain at the α+/β− interface was demonstrated to be the target for the pyrazoloquinoline CGS 9895 (2-p-methoxyphenylpyrazolo[4,3-c]quinolin-3-(5H)-one, as shown in Figure 4), which was already identified as a null modulator (antagonist) at the high-affinity benzodiazepine site, and acts as a positive allosteric modulator [27]. Therefore, compound **6g** was further tested for its ability to antagonize the potentiation of the GABA_A_ receptor induced by CGS 9895.

The data obtained in GABA_A_ receptors devoid of the γ subunit (α_1_β_2_) indicates that compound **6g** reduces the potentiation of the GABA_A_ receptor induced by CGS 9895 (10 μM) (about −58% at both 10 μM and 30 μM), suggesting that this compound binds the α+/β− low-affinity site, too. This interesting result seems to suggest that our compounds acting as null modulators or antagonists could be selective α+/β− low-affinity benzodiazepine binding site ligands. This hypothesis was preliminarily confirmed by compounds **5d** and **5e**, which in the same test completely blocked or strongly reduced the potentiation of the GABA_A_ receptor function induced by CGS 9895, respectively (data not shown). In accordance with Sieghart et al. (2012) [8], we believe that drugs acting at the “non-canonical” α+/β− low-affinity binding site might display potential clinical relevance. It has been proposed that the compounds acting at this site could be beneficial for long-term epilepsy treatment. In fact, they will be able to interact with a broader variety of GABA_A_ receptors a subtypes such as the δ, ε, and π subunit-containing GABA_A_ receptors.

Results from electrophysiological studies on recombinant α1β2γ2L GABA_A_ receptors showed that the 3-aroyl derivatives are able to slightly induce a change in the agonist-evoked current. This evidence led us to speculate that the aroyl moiety at position 3 of the PQ scaffold could be responsible for this effect. The more active compounds **6a** and **6b** respectively bear the 2-methoxybenzoyl and thien-2-yl-carbonyl groups at position 3, which are both able to engage hydrogen bond interaction with receptor proteins. Also, it is noteworthy that compound 6b retains the same pyrazolo[1,5-a]pyrimidine substructure as indiplon (Figure 1), which is already known as a partial agonist. In particular, the carbonyl moiety (CO) and the 2-methoxy (2-OMe) substituent on the phenyl ring for **6a** and the sulfur atom of the thienyl ring for **6b** could provoke a change of conformation favorable for the effect; this is different from the 3-aryl substituted PQ, which is devoid of activity.

The very interesting result obtained with the 3-(4-methoxyphenylcarbonyl)-8-methoxy-4,5-dihydropyrazolo[1,5-a]quinazoline **11d**, that shows an enhanced current at 1 μM, encouraged us to synthesize other derivatives with the 4,5-dihydropyrazolo[1,5-a]quinazoline scaffold, which is certainly worthy of remark.

## 3. Material and Methods

Melting points were determined with a Gallenkamp apparatus and were uncorrected. Silica gel plates (Merk F_254_) and silica gel 60 (Merk 70–230 mesh) were used for analytical and column chromatography, respectively. The structures of all the compounds were supported by their IR spectra (KBr pellets in nujol mulls, Perkin-Elmer 1420 spectrophotometer) and ^1^H-NMR data (measured with a Bruker 400 MHz). Chemical shifts were expressed in δ ppm, using DMSO-d_6_ or CDCl_3_ as solvent. The chemical and physical data of new compounds are shown in Table S1; all of the microanalyses were performed with a Perkin-Elmer 260 analyzer for C, H, and N. Mass spectra (*m*/*z*) were recorded on an Electrospray ionisation time-of-flight mass spectrometry ESI-TOF mass spectrometer (Bruker Micro TOF), and reported mass values are within the error limits of ±5 ppm mass units. Microanalyses indicated by the symbols of the elements or functions were performed with a Perkin-Elmer 260 elemental analyzer for C, H, and N, and they were within ±0.4% of the theoretical values.

### 3.1. General Procedure for the Synthesis of ***3b**–**e***

A suspension of 2-hydrazinobenzoic acid (**1**) or 2-hydrazino-4-methoxybenzoic acid (**2**) (1.0 mmol) and 3-oxo-2-(2-thienyl)-propionitrile, 3-oxo-2-(3-thienyl)-propionitrile (1.1 mmol) in acetic acid (5 mL) was refluxed until the starting material disappeared in TLC (toluene/ethyl acetate/acetic acid 8:2:1 *v*/*v*/*v* as eluent). The final solution was treated with ice/water, and the precipitate was filtered and purified by recrystallization by a suitable solvent.

#### 3.1.1. 3-(Thien-2-yl)pyrazolo[1,5-a]quinazolin-5(4H)-one (**3b**)

From **1** and 3-oxo-2-(2-thienyl)-propionitrile. Cream crystals, yield 80%; IR ν cm^−1^ 3120, 1674; ^1^H-NMR (DMSO-d_6_) δ 12.07 (bs, 1H, NH, exch.); 8.14 (d, 1H, H-9, *J* = 8.0); 8.09 (d, 1H, H-6, *J* = 8.0); 7.99 (s, 1H, H-2); 7.89 (t, 1H, H-7, *J* = 7.8 Hz); 7.50 (t, 1H, H-8, *J* = 7.8 Hz); 7.46 (d, 1H, H-3 thienyl, *J* = 4.8 Hz); 7.38 (d, 1H, H-5 thienyl, *J* = 3.6); 7.11 (m, 1H, H-4 thienyl). ESI-MS calcd for C_14_H_9_N_3_OS (267.31); found: *m*/*z* 268.01[M + H]^+^. Anal C_14_H_9_N_3_OS (C, H, N).

#### 3.1.2. 3-(Thien-3-yl)pyrazolo[1,5-a]quinazolin-5(4H)-one (**3c**)

From **1** and 3-oxo-2-(3-thienyl)-propionitrile. Cream crystals, yield 52%; IR ν cm^−1^ 3120, 1674; ^1^H-NMR (CDCl_3_) δ 9.22 (bs, 1H, NH, exch.); 8.29 (d, 1H, H-9, *J* = 8.0); 8.20 (d, 1H, H-6, *J* = 8.0); 7.88 (s, 1H, H-2); 7.81 (t, 1H, H-7, *J* = 7.8 Hz); 7.48 (m, 2H, H-8, and H-2 thienyl); 7.35 (m, 2H, H-4, and H-5 thienyl). ESI-MS calcd for C_14_H_9_N_3_OS (267.31); found: *m*/*z* 267.88[M + H]^+^. Anal C_14_H_9_N_3_OS (C, H, N).

#### 3.1.3. 8-Methoxy-3-(thien-2-yl)pyrazolo[1,5-a]quinazolin-5(4H)-one (**3d**)

From **2** and 3-oxo-2-(2-thienyl)-propionitrile. Cream crystals, yield 90%; IR ν cm^−1^ 3137, 1697; ^1^H-NMR (DMSO-d_6_) δ 11.89 (bs, 1H, NH, exch.); 8.05 (d, 1H, H-6, *J* = 8.8 Hz); 7.98 (s, 1H, H-2); 7.51 (d, H-9, *J* = 2.0 Hz); 7.46 (d, 1H, H-3 thienyl, *J* = 4.8 Hz); 7.38 (d, 1H, H-5 thienyl, *J* = 3.4 HZ); 7.09 (dd, 1H, H-4 thienyl, *J* = 4.8 Hz, *J* = 3.4 Hz); 7.06 (dd, 1H, H-7, *J* = 8.8 Hz, *J* = 2.0 Hz); 3.94 (s, 3H, OCH_3_). ESI-MS calcd for C_15_H_11_N_3_O_2_S (297.33); found: *m*/*z* 297.93[M + H]^+^. Anal. C_15_H_11_N_3_O_2_S (C, H, N).

#### 3.1.4. 8-Methoxy-3-(thien-3-yl)pyrazolo[1,5-a]quinazolin-5(4H)-one (**3e**)

From **2** and 3-oxo-2-(3-thienyl)-propionitrile. Cream crystals, yield 67%; IR ν cm^−1^ 3110, 1687; ^1^H-NMR (DMSO-d_6_) δ 11.78 (bs, 1H, NH, exch.); 8.16 (s, 1H, H-2); 8.05 (d, 1H, H-6, *J* = 9.0 Hz); 7.84 (m, 1H, H-2 thienyl); 7.59 (m, 1H, H-4 thienyl); 7.51 (d, H-9, *J* = 2.4 Hz); 7.49 (d, 1H, H-5 thienyl, *J* = 4.0 Hz); 7.05 (dd, 1H, H-7, *J* = 9.0 Hz, *J* = 2.4 Hz); 3.94 (s, 3H, OCH_3_). ESI-MS calcd for C_15_H_11_N_3_O_2_S (297.33); found: *m*/*z* 298.03[M + H]^+^. Anal. C_15_H_11_N_3_O_2_S (C, H, N).

### 3.2. General Procedure for the Synthesis of ***3f**–**h***

A suspension of 2-hydrazino-4-methoxybenzoic acid (**2**, 1.09 mmol), 2-aroyl-3-(dimethylamino) acrylonitrile (1.09 mmol), and anhydrous sodium acetate (1.31 mmol) in DMF (4 mL) was refluxed until the starting material disappeared in TLC (toluene/ethyl acetate/methanol 8:2:1.5 *v*/*v*/*v* as eluent). The final solution was treated with ice/water, and the precipitate was filtered and purified by recrystallization by a suitable solvent.

#### 3.2.1. 8-Methoxy-3-(2-methoxybenzoyl)pyrazolo[1,5-a]quinazolin-5(4H)-one (**3f**)

From **2** and 2-benzoyl-3-(dimethylamino)acrylonitrile. Cream crystals, yield 50%; IR ν cm^−1^ 3167, 1687, 1674; ^1^H-NMR (DMSO-d_6_) δ 11.29 (bs, 1H, NH, exch.); 8.09 (d, 1H, H-6, *J* = 8.8 Hz); 7.86 (s, 1H, H-2); 7.52 (t, 1H, H-4 phenyl, *J* = 8.4 Hz); 7.49 (d, H-9, *J* = 2.0 Hz); 7.38 (d, 1H, H-6 phenyl, *J* = 6.4 Hz); 7.19 (d, 1H, H-3 phenyl, *J* = 8.4 Hz); 7.13 (dd, 1H, H-7 *J* = 8.8 Hz, *J* = 2.0 Hz); 7.06 (t, 1H, H-5 phenyl, *J* = 7.2 Hz); 3.94 (s, 3H, 8-OCH_3_); 3.77 (s, 3H, OCH_3_). ESI-MS calcd for C_19_H_15_N_3_O_4_ (349,34); found: *m*/*z* 349.94[M + H]^+^. Anal. C_19_H_15_N_3_O_4_ (C, H, N).

#### 3.2.2. 8-Methoxy-3-(thien-2-ylcarbonyl)pyrazolo[1,5-a]quinazolin-5(4H)-one (**3g**)

From **2** and 2-(2-thienylcarbonyl)-3-(dimethylamino)acrylonitrile. Pale yellow crystals, yield 65%; IR ν cm^−1^ 3327, 1699, 1678; ^1^H-NMR (DMSO-d_6_) δ 11.23 (bs, 1H, NH, exch.); 8.62 (s, 1H, H-2); 8.13–8.05 (m, 3H, H-6 and H-3, H-5 thienyl); 7.53 (d, 1H, H-9 *J* = 2.0 Hz); 7.30 (m, 1H, H-4 thienyl); 3.95 (s, 3H, OCH_3_). ESI-MS calcd for C_16_H_11_N_3_O_3_S (325,34); found: *m*/*z* 326.04[M + H]^+^. Anal. C_16_H_11_N_3_O_3_S (C, H, N).

#### 3.2.3. 8-Methoxy-3-(thien-3-ylcarbonyl)pyrazolo[1,5-a]quinazolin-5(4H)-one (**3h**)

From **2** and 2-(3-thienylcarbonyl)-3-(dimethylamino)acrylonitrile. Pale yellow crystals, yield 65%; IR ν cm^−1^ 3310, 1698, 1678; ^1^H-NMR (DMSO-d_6_) δ 11.24 (bs, 1H, NH, exch.); 8.54 (d, 1H, H-2 thienyl, *J* = 1.2 Hz); 8.48 (s, 1H, H-2); 8.10 (d, 1H, H-6, *J* = 8.8 Hz); 7.71 (dd, 1H, H-5 thienyl, *J* = 5.2 Hz, *J* = 2.8 Hz); 7.59 (d, 1H, H-4 thienyl, *J* = 4.8 Hz); 7.54 (d, H-9, *J* = 2.0 Hz); 7.15 (dd, 1H, H-7, *J* = 8.8 Hz, *J* = 2.0 Hz); 3.96 (s, 3H, OCH_3_). ESI-MS calcd for C_16_H_11_N_3_O_3_S (325,34); found: *m*/*z* 325.94[M + H]^+^. Anal. C_16_H_11_N_3_O_3_S (C, H, N).

### 3.3. General Procedure for the Synthesis of ***4a**–**c***

A solution of **3f**–**h** (0.3 mmol) in POCl_3_ (3 mL) was heated (40–80 °C) and maintained under stirring until the starting material disappeared, as evaluated by TLC (toluene/ethyl acetate/methanol 8:2:1.5 *v*/*v*/*v* as eluent). The final solution was evaporated, and the residue was recuperated with ice/water, filtered, and washed with water and ethanol 80%.

#### 3.3.1. 3-(2-Methoxyphenylcarbonyl)-5-chloro-8-methoxypyrazolo[1,5-a]quinazoline (**4a**)

Pale cream crystals, yield 95%; IR ν cm^−1^ 1674; ^1^H-NMR (DMSO-d_6_) δ 8.44 (s, 1H, H-2); 8.19 (d, 1H, H-6, *J* = 9.2 Hz); 7.80 (d, H-9, *J* = 2.0 Hz); 7.52 (t, 1H, H-4 phenyl, *J* = 8.4 Hz); 7.36 (d, 2H, H-6 phenyl and H-3 phenyl, *J* = 6.8 Hz); 7.15 (d, 1H, H-7 *J* = 8.4 Hz); 7.04 (t, 1H, H-5 phenyl, *J* = 7.2 Hz); 4.04 (s, 3H, 8-OCH_3_); 3.64 (s, 3H, OCH_3_). ESI-MS calcd for C_19_H_14_N_3_O_3_Cl (367,79); found: *m*/*z* 368.39[M + H]^+^. Anal. C_19_H_14_N_3_O_3_Cl (C, H, N).

#### 3.3.2. 3-(Thien-2-ylcarbonyl)-5-chloro-8-methoxypyrazolo[1,5-a]quinazoline (**4b**)

Pale yellow crystals, yield 95%; IR ν cm^−1^ 1648; ^1^H-NMR (DMSO-d_6_) δ 8.72 (s, 1H, H-2); 8.22 (d, 1H, H-6, *J* = 9.2 Hz); 8.13 (d, 1H, H-3 thienyl *J* = 2.8 Hz); 8.06 (d, 1H, H-5 thienyl, *J* = 4.4 Hz); 7.82 (d, 1H, H-9 *J* = 2.0 Hz); 7.36 (dd, 1H, H-7, *J* = 8.8 Hz, *J* = 2.0 Hz); 7.29 (m, 1H, H-4 thienyl); 4.05 (s, 3H, OCH_3_). ESI-MS calcd for C_16_H_10_N_3_O_2_SCl (343,79); found: *m*/*z* 344.49[M + H]^+^. Anal. C_16_H_10_N_3_O_2_SCl (C, H, N).

#### 3.3.3. 3-(Thien-3-ylcarbonyl)-5-chloro-8-methoxypyrazolo[1,5-a]quinazoline (**4c**)

Pale yellow crystals, yield 75%; IR ν cm^−1^ 1647; ^1^H-NMR (DMSO-d_6_) δ 8.66 (s, 1H, H-2); 8.48 (d, 1H, H-2 thienyl, *J* = 1.2 Hz); 8.23 (d, 1H, H-6, *J* = 8.8 Hz); 7.85 (d, H-9, *J* = 2.0 Hz); 7.66 (m, 1H, H-5 thienyl); 7.59 (d, 1H, H-4 thienyl, *J* = 4.8 Hz); 7.38 (dd, 1H, H-7, *J* = 8.8 Hz, *J* = 2.0 Hz); 4.06 (s, 3H, OCH_3_). ESI-MS calcd for C_16_H_10_N_3_O_2_SCl (343,79); found: *m*/*z* 344.49[M + H]^+^. Anal. C_16_H_10_N_3_O_2_SCl (C, H, N).

### 3.4. General Procedure for the Synthesis of ***5a**–**e***

LiAlH_4_/THF solution (2.8 mmol) was added to a suspension of 3**a** [25] and 3**b**–**e** (1.0 mmol) in anhydrous THF and the mixture refluxed. The reaction was monitored with TLC (toluene/ethyl acetate/methanol 8:2:1.5 *v*/*v*/*v* as eluent) until the starting material disappeared and a mixture of compounds was formed: the 4,5-dihydro and the 4,5-dehydro derivatives. Thus, the reaction was maintained at air to permit the complete oxidation to final pyrazolo[1,5-a]quinazoline derivatives. The careful addition of water, the next extraction with ethyl acetate, and the followed evaporation gave the final desired compounds.

#### 3.4.1. 3-Phenylpyrazolo[1,5-a]quinazoline (**5a**)

Yellow crystals, yield 75%; ^1^H-NMR (DMSO-d_6_) δ 9.24 (s, 1H, H-5); 8.84 (d, 1H, H-9, *J* = 8.0 Hz); 8.67 (s, 1H, H-2); 8.29 (d, 2H, H-2 phenyl and H-6 phenyl, *J* = 8.0 Hz); 8.24–8.19 (m, 2H, H-6, and H-4 phenyl); 7.86 (t, 1H, H-8, *J* = 8.0 Hz); 7.73 (t, 2H, H-3 phenyl and H-5 phenyl, *J* = 8.0 Hz); 7.56 (t, 1H, H-7, *J* = 8.0 Hz). ^13^C-NMR (DMSO-d_6_) δ 155.7, 131.9, 130.8, 129.1, 128.7, 127.5, 97.5. ESI-MS calcd for C_16_H_11_N_3_ (245,10); found: *m*/*z* 245.8[M + H]^+^. Anal. C_16_H_11_N_3_ (C, H, N).

#### 3.4.2. 3-(Thien-2-yl)pyrazolo[1,5-a]quinazoline (**5b**)

Pale yellow crystals, yield 52%; ^1^H-NMR (DMSO-d_6_) δ 9.08 (s, 1H, H-5); 8.62 (s, 1H, H-2); 8.37 (d, 1H, H-9, *J* = 8.0); 8.21 (d, 1H, H-6, *J* = 8.0); 7.82–7.63 (m, 2H, H-8, and H-5 thienyl); 7.46 (d, 1H, H-3 thienyl *J* = 4.6 Hz); 7.25 (t, 1H, H-7, *J* = 8.8 Hz); 7.27 (dd, 1H, H-4 thienyl, *J* = 4.6 Hz, *J* = 3.8). ^13^C-NMR (DMSO-d6) δ 165.5, 154.9, 145.7, 137.7, 135.4 135.1, 135.0, 131.8, 129.1, 120.8, 97.5. ESI-MS calcd for C_15_H_11_N_3_OS (281,33); found: m/z 281.93[M + H]+. Anal. C_15_H_11_N_3_OS (C, H, N).

#### 3.4.3. 3-(Thien-3-yl)pyrazolo[1,5-a]quinazoline (**5c**)

Yellow crystals, yield 47%; ^1^H-NMR (DMSO-d_6_) δ 9.16 (s, 1H, H-5); 8.64 (s, 1H, H-2); 8.37 (d, 1H, H-9, *J* = 8.0); 8.21 (d, 1H, H-6, *J* = 8.0); 8.04–7.99 (m, 2H, H-8, and H-2 thienyl); 7.84 (d, 1H, H-5 thienyl, *J* = 4.8 Hz); 7.67 (m, 2H, H-7, and H-4 thienyl). ^13^C-NMR (DMSO-d_6_) δ 155.7, 133.4, 132.3, 128.3, 127.7, 127.4, 124.7, 76.8. ESI-MS calcd for C_14_H_9_N_3_S (251,31); found: *m*/*z* 252.71[M + H]^+^. Anal. C_14_H_9_N_3_S (C, H, N).

#### 3.4.4. 3-(Thien-2-yl)-8-methoxypyrazolo[1,5-a]quinazoline (**5d**)

Pale yellow crystals, yield 75%; ^1^H-NMR (DMSO-d_6_) δ 9.05 (s, 1H, H-5); 8.59 (s, 1H, H-2); 8.14 (d, 1H, H-6 *J* = 8.8 Hz); 7.75 (d, 1H, H-9 *J* = 2.0 Hz); 7.63 (d, 1H, H-5 thienyl, *J* = 3.8 Hz); 7.46 (d, 1H, H-3 thienyl *J* = 4.6 Hz); 7.25 (dd, 1H, H-7, *J* = 8.8 Hz, *J* = 2.0 Hz); 7.27 (dd, 1H, H-4 thienyl, *J* = 4.6 Hz, *J* = 3.8); 4.01 (s, 3H, OCH_3_). ^13^C-NMR (DMSO-d_6_) δ 165.5, 154.9, 145.7, 137.7, 135.4 135.1, 135.0, 131.8, 129.1, 120.8, 117.5, 106.4, 55.8. ESI-MS calcd for C_15_H_11_N_3_OS (281,33); found: *m*/*z* 281.93[M + H]^+^. Anal. C_15_H_11_N_3_OS (C, H, N).

#### 3.4.5. 3-(Thien-3-yl)-8-methoxypyrazolo[1,5-a]quinazoline (**5e**)

Pale yellow crystals, yield 70%; ^1^H-NMR (DMSO-d_6_) δ 9.04 (s, 1H, H-5); 8.62 (s, 1H, H-2); 8.13 (d, 1H, H-6, *J* = 8.8 Hz); 8.03 (d, 1H, H-2 thienyl *J* = 2.8 Hz); 7.85 (dd, 1H, H-4 thienyl, *J* = 5.0 Hz, *J* = 1.2 Hz); 7.75 (d, H-9, *J* = 2.2 Hz); 7.63 (dd, 1H, H-5 thienyl *J* = 5.0 Hz, *J* = 3.0 Hz); 7.24 (dd, 1H, H-7, *J* = 8.8 Hz, *J* = 2.2 Hz); 4.01 (s, 3H, OCH_3_). ^13^C-NMR (DMSO-d_6_) δ 165.9, 155.4, 145.5, 136.7, 128.9, 127.6, 125.2, 120.8, 117.5, 106.4, 55.8. ESI-MS calcd for C_15_H_11_N_3_OS (281,33); found: *m*/*z* 281.93[M + H]^+^. Anal. C_15_H_11_N_3_OS (C, H, N).

### 3.5. General Procedure for the Synthesis of ***5f**–**h***

Triphenylphosphine palladium (0) (Tetrakis, 0.035 mmol), 2.4 mL of Na_2_CO_3_ solution 2M, and suitable (hetero)arylboronic acid (0.60 mmol) solubilized in 1 mL of absolute ethanol were added to a solution of 3-iodo- or 3-bromo-8-methoxypyrazolo[1,5-a]quinazoline (**9a**, **9b**, see below) (0.2 mmol) in anhydrous THF (6 mL). The suspension was refluxed until the starting material disappeared in TLC; then, it was diluted with ethyl acetate, and water was added. The organic layer was separated and dried over Na_2_SO_4_ anhydrous and evaporated to dryness. The final compounds were purified by recrystallization by a suitable solvent. In general, it has been observed that from starting material **9a**, the yield of coupling is better than starting from **9b**.

#### 3.5.1. 3-(2-Methoxyphenyl)-8-methoxypyrazolo[1,5-a]quinazoline (**5f**)

From **9a** and 2-methoxyphenylboronic acid. Cream crystals, yield 45% (from **9b** yield 20%); TLC:cyclohexane/ethyl acetate 2:1 *v*/*v*; ^1^H-NMR (CDCl_3_) δ 8.82 (s, 1H, H-5); 8.61 (s, 1H, H-2); 8.20 (dd, 1H, H-7, *J* = 8.2 Hz, *J* = 2.0 Hz); 7.87 (d, H-9, *J* = 2.0 Hz); 7.83 (d, 1H, H-6, *J* = 8.2 Hz); 7.30 (dt, 1H, H-4 phenyl, *J* = 8.4 Hz, *J* = 1.6 Hz); 7.13–7.08 (m, 2H, H-5, H-6 phenyl); 7.03 (d, 1H, H-3 phenyl, *J* = 8.4 Hz); 4.05 (s, 3H, 8-OCH_3_); 3.92 (s, 3H, OCH_3_). ^13^C-NMR (DMSO-d_6_) δ 165.3, 160.9, 155.0, 145.6, 145.2, 137.7, 133.6, 131.7, 126.6, 123.9, 123.7, 117.5, 114.4, 113.8, 104.6, 96.3, 56.8, 55.8. ESI-MS calcd for C_18_H_15_N_3_O_2_ (305,33); found: *m*/*z* 305.93[M + H]^+^. Anal. C_18_H_15_N_3_O_2_ (C, H, N).

This compound was the only one product recovered by treatment of **10** with SOCl_2_ and 2-MeO-PhB(OH)_2_ in toluene and Na_2_CO_3_ sol. 2 M, in the attempt to obtain the 3-(2-methoxyphenylcarbonyl)-8-methoxypyrazolo[1,5-a]quinazoline **6a**, yield 15%.

#### 3.5.2. 3-(Fur-3-yl)-8-methoxypyrazolo[1,5-a]quinazoline (**5g**)

From **9a** and 3-furylboronic acid. Pale yellow crystals, yield 43% (from **9b** yield 22%); TLC: CHX/ethyl acetate 4:1 *v*/*v*; ^1^H-NMR (DMSO-d_6_) δ 8.97 (s, 1H, H-5); 8.47 (s, 1H, H-2); 8.13 (m, 2H, H-6 and H-4 furyl); 7.27 (m, 2H, H-2 furyl and H-9); 7.21 (dd, 1H, H-7, *J* = 8.8 Hz, *J* = 2.2 Hz); 7.10 (s, 1H, H-5 furyl); 3.98 (s, 3H, OCH_3_). ^13^C-NMR (DMSO-d_6_) δ 155.4, 144.1, 138.5, 133.4, 128.9, 120.8, 108.8, 106.4, 76.8, 55.8. ESI-MS calcd for C_15_H_11_N_3_O_2_ (265,27); found: *m*/*z* 265.97[M + H]^+^. Anal. C_15_H_11_N_3_O_2_ (C, H, N).

#### 3.5.3. Tert-Butyl 2-(8-methoxypyrazolo[1,5-a]quinazolin-3-yl)-1H-pyrrole-1-carboxylate (**5h**)

From **9a** and N-BOC-pyrrol-2-boronic acid. Oil, yield 37%; (from **9b** yield 18%); TLC: toluene/ethyl acetate/acetic acid 8:2:1 *v*/*v*/*v*; ^1^H-NMR (CDCl_3_) δ 8.77 (s, 1H, H-5); 8.18 (s, 1H, H-2); 8.13 (m, 2H, H-6, and H-9); 7.41 (dd, 1H, H-3 pyrrole, *J* = 3.2 Hz, *J* = 1.6 Hz); 7.10 (dd, 1H, H-7, *J* = 8.8 Hz, *J* = 2.4 Hz); 6.46 (d, 1H, H-5 pyrrole, *J* = 2.8 Hz); 4.02 (s, 3H, OCH_3_); 1.35 (s, 9H, t-Bu). ^13^C-NMR (DMSO-d_6_) δ 155.4, 150.2, 133.4, 128.9, 120.8, 119.2, 115.9, 106.4, 99.0, 84.2, 55.8, 28.4. ESI-MS calcd for C_20_H_20_N_4_O_3_ (364.40); found: *m*/*z* 365.0[M + H]^+^. Anal. C_20_H_20_N_4_O_3_ (C, H, N).

### 3.6. 3-(1H-Pyrrol-2-yl)-8-methoxypyrazolo[1,5-a]quinazoline (***5i***)

A solution of **5h** (0.11 mmol) in 2 mL of anhydrous THF was added to 1.5 mL of sodium methoxide solution 0.43 M. The reaction was kept at 40 °C for 4 h; then, it was cooled and diluted with ice/water until a precipitate was formed. Then, it was filtered and recrystallized by ethanol; yield 69%. TLC:CHX/ethyl acetate 4:1 *v*/*v*; ^1^H-NMR (CDCl_3_) δ 10.32 (bs, 1H, NH, exch.); 8.73 (s, 1H, H-5); 8.30 (s, 1H, H-2); 7.84 (m, 2H, H-6 and H-9); 7.11 (dd, 1H, H-7, *J* = 8.8 Hz, *J* = 2.0 Hz); 6.93 (m, 1H, H-3 pyrrole); 6.54 (m, 1H, H-5 pyrrole); 6.30 (m. 1H, H-4 pyrrole); 4.05 (s, 3H, OCH_3_). ^13^C-NMR (CDCl_3_) δ 165.8, 155.4, 146.8, 133.4, 120.8, 120.2, 124.0, 111.5, 106.8, 106.4, 55.8. ESI-MS calcd for C_15_H_12_N_4_O (264.28); found: *m*/*z* 264.88[M + H]^+^. Anal. C_15_H_12_N_4_O (C, H, N).

### 3.7. General Procedure for the Synthesis of ***6a**–**c***

Ammonium formate 97% (7.83 mmol) and 20 mg of the catalyst Pd/C 10% was added to a solution of **4a**–**c** (0.3 mmol) in ethanol abs. (4 mL). The reaction was maintained at reflux temperature for 1 h; then, the filtration of the catalyst and the next evaporation of the solution gave a residue that was recovered by ethanol 80%.

#### 3.7.1. 3-(2-Methoxyphenylcarbonyl)-8-methoxypyrazolo[1,5-a]quinazoline (**6a**)

Pale cream crystals, yield 68%; IR ν cm^−1^ 1639; ^1^H-NMR (DMSO-d_6_) δ 9.11 (s, 1H, H-5); 8.45 (s, 1H, H-2); 8.18 (d, 1H, H-6, *J* = 8.8 Hz); 7.79 (d, H-9, *J* = 2.0 Hz); 7.50 (t, 1H, H-4 phenyl, *J* = 8.4 Hz); 7.36–7.31 (m, 2H, H-7, and H-6 phenyl); 7.13 (d, 1H, H-3 phenyl, *J* = 8.4 Hz); 7.03 (t, 1H, H-5 phenyl, *J* = 7.6 Hz); 4.03 (s, 3H, 8-OCH_3_); 3.61 (s, 3H, OCH_3_). ^13^C-NMR (DMSO-d_6_) δ 196.3, 165.3, 160.9, 155.0, 145.6, 145.2, 137.7, 133.6, 131.7, 126.6, 123.9, 123.7, 117.5, 114.4, 113.8, 104.6, 96.3, 56.8, 55.8. ESI-MS calcd for C_19_H_15_N_3_O_3_ (333,34); found: *m*/*z* 333.94[M + H]^+^. Anal. C_19_H_15_N_3_O_3_ (C, H, N).

#### 3.7.2. 3-(Thien-2-ylcarbonyl)-8-methoxypyrazolo[1,5-a]quinazoline (**6b**)

Pale yellow crystals, yield 74%; IR cm^−1^ 1640; ^1^H-NMR (DMSO-d_6_) δ 9.22 (s, 1H, H-5); 8.70 (s, 1H, H-2); 8.21 (m, 2H, H-6 and H-3 thienyl); 8.03 (d, 1H, H-5 thienyl, *J* = 4.8 Hz); 7.82 (d, 1H, H-9, *J* = 2.0 Hz); 7.34 (dd, 1H, H-7, *J* = 8.8 Hz, *J* = 2.0 Hz); 7.27 (dd, 1H, H-4 thienyl, *J* = 4.4 Hz, *J* = 4.0); 4.03 (s, 3H, OCH_3_). ^13^C-NMR (DMSO-d_6_) δ 179.5, 165.5, 154.9, 145.7, 137.7, 135.4 135.1, 135.0, 131.8, 129.1, 120.8, 117.5, 106.4, 55.8. ESI-MS calcd for C_16_H_11_N_3_O_2_S (309,34); found: *m*/*z* 309.94[M + H]^+^. Anal. C_16_H_11_N_3_O_2_S (C, H, N).

#### 3.7.3. 3-(Thien-3-ylcarbonyl)-8-methoxypyrazolo[1,5-a]quinazoline (**6c**)

Pale yellow crystals, yield 70%; IR ν cm^−1^ 1640; ^1^H-NMR (DMSO-d_6_) δ 9.22 (s, 1H, H-5); 8.70 (s, 1H, H-2); 8.49 (s, 1H, H-2 thienyl); 8.20 (d, 1H, H-6, *J* = 8.8 Hz); 7.82 (d, H-9, *J* = 2.0 Hz); 7.66 (m, 1H, H-5 thienyl); 7.59 (d, 1H, H-4 thienyl, *J* = 4.8 Hz); 7.34 (dd, 1H, H-7, *J* = 8.8 Hz, *J* = 2.0 Hz); 4.04 (s, 3H, OCH_3_). ^13^C-NMR (DMSO-d_6_) δ 192.1, 165.9, 155.4, 145.5, 136.7, 128.9, 127.6, 125.2, 120.8, 117.5, 106.4, 55.8. ESI-MS calcd for C_16_H_11_N_3_O_2_S (309,34); found: *m*/*z* 309.94[M + H]^+^. Anal. C_16_H_11_N_3_O_2_S (C, H, N).

### 3.8. 3-(4-Methoxyphenylcarbonyl)-8-methoxypyrazolo[1,5-a]quinazoline (***6d***)

A 100-mL round-bottomed flask was charged with 8-methoxypyrazolo[1,5-a] quinazoline-3-carboxylic acid, **10** [18] (0.3 mmol), and the commercially available Eaton’s reagent (7.7%, *w*/*w*, of P_2_O_5_ in MsOH) (2.5 equiv) was added. The solution was kept at 60 °C for 20 min. After that, anisole (0.35 mmol) was added and maintained for 2 h at 60 °C. The final solution was treated with saturated solution of NaHCO_3_ to reach pH 8; the obtained precipitate was filtered and purified by recrystallization. Pale cream crystals, yield 66%; IR ν cm^−1^ 1639; ^1^H-NMR (DMSO-d_6_) δ 9.13 (s, 1H, H-5); 8.56 (s, 1H, H-2); 8.20 (d, 1H, H-6, *J* = 8.8 Hz); 7.88 (d, 2H, H-2 and H-6 phenyl, *J* = 8.0 Hz); 7.82 (d, 1H, H-9, *J* = 2.0 Hz); 7.33 (dd, 1H, H-7, *J* = 8.0 Hz, *J* = 2.2 Hz); 7.04 (d, 2H, H-3 and H-5 phenyl, *J* = 8.0 Hz); 4.03 (s, 3H, 8-OCH_3_); 3.84 (s, 3H, OCH_3_). ^13^C-NMR (DMSO-d_6_) δ 187.1, 165.3, 163.2, 154.6, 154.5, 145.9, 144.6, 137.7, 132.3, 131.7, 131.6, 129.2, 120.7, 117.5, 117.2, 114.0, 113.8, 113.6, 112.9, 112.2, 96.4, 56.8, 55.9. ESI-MS calcd for C_19_H_15_N_3_O_3_ (333,34); found: *m*/*z* 333.94[M + H]^+^. Anal. C_19_H_15_N_3_O_3_ (C, H, N).

### 3.9. General Procedure for The Synthesis of ***6e**–**g***

The starting material 8-methoxypyrazolo[1,5-a]quinazoline-3-carboxylic acid, **10** [18] (0.3 mmol), was suspended in CH_2_Cl_2_, 0.15 mL of trichloroacetonitrile, and 1.45 mmol of PPh_3_. The mixture was maintained at room temperature for 2 h, monitoring the reaction by TLC; when the corresponding 3-carbonylchloride was formed, SnCl_4_ anhydrous (0.9 mL) and the suitable heteroaryl were added. After 2 h, the reaction was quenched by the addition of HCl 6M, and the final solution was extracted with ethyl acetate. The organic layer was washed with NaOH 10% solution, dried, and evaporated, obtaining the final compound that was purified by recrystallization.

#### 3.9.1. 3-(Fur-2-ylcarbonyl)-8-methoxypyrazolo[1,5-a]quinazoline (**6e**)

From **10** and furane. Pale yellow crystals, yield 20%; TLC: toluene/ethyl acetate/acetic acid 8:2:2 *v*/*v*/*v*; IR ν cm^−1^ 1630; ^1^H-NMR (DMSO-d_6_) δ 9.25 (s, 1H, H-5); 8.80 (s, 1H, H-2); 8.24 (d, 1H, H-6, *J* = 8.8 Hz); 8.06 (s, 1H, H-5 furyl); 7.83 (d, 1H, H-9 *J* = 2.0 Hz); 7.66 (d, 1H, H-3 furyl, *J* = 3.6 Hz); 7.35 (dd, 1H, H-7, *J* = 8.8 Hz, *J* = 2.0 Hz); 6.77 (s, 1H, H-4 furyl); 4.04 (s, 3H, OCH_3_). ^13^C-NMR (DMSO-d_6_) δ 174.5, 154.2, 145.7, 138.0, 130.3, 118.7, 117.7, 113.9, 112.3, 111.8, 96.2, 56.4. ESI-MS calcd for C_16_H_11_N_3_O_3_ (293.28); found: *m*/*z* 293.88[M + H]^+^. Anal. C_16_H_11_N_3_O_3_ (C, H, N).

#### 3.9.2. 3-(1H-pyrrol-2-ylcarbonyl)-8-methoxypyrazolo[1,5-a]quinazoline (**6f**)

From **10** and pyrrole. Cream crystals, yield 25%; TLC: toluene/ethyl acetate/acetic acid 8:2:2 *v*/*v*/*v*; ^1^H-NMR (CDCl_3_) δ 12.31 (bs, 1H, NH, exch.); 9.10 (s, 1H, H-5); 8.81 (s, 1H, H-2); 7.96 (d, 1H, H-6, *J* = 8.8 Hz); 7.90 (d, 1H, H-9, *J* = 2.0 Hz); 7.37 (m, 1H, H-3 pyrrole); 7.22 (dd, 1H, H-7, *J* = 8.8 Hz, *J* = 2.0 Hz); 7.16 (m, 1H, H-5 pyrrole); 6.39 (m. 1H, H-4 pyrrole); 4.08 (s, 3H, OCH_3_). ^13^C-NMR (CDCl_3_) δ 175.0, 165.8, 152.7, 146.8, 138.5, 130.5, 124.0, 117.8, 117.7, 113.9, 113.2, 110.9, 96.1, 56.4. ESI-MS calcd for C_16_H_12_N_4_O_2_ (292.29); found: *m*/*z* 292.99[M + H]^+^. Anal. C_16_H_12_N_4_O_2_ (C, H, N).

#### 3.9.3. 3-(1-Methyl-1H-pyrrol-2-ylcarbonyl)-8-methoxypyrazolo[1,5-a]quinazoline (**6g**)

This product was obtained following the Friedel–Craft reaction from **10** and 1-methyl-1*H*-pyrrole, yield 10%; or by the alkylation of **6f** following the classical alkylation method: 0.15 mmol of **6f** in 3 mL of anhydrous DMF, 0.15 mmol of NaH, and 0.02 mL of MeI. The reaction was kept at 35 °C for 1 h, and the precipitate that was obtained by the addition of water was filtered and recrystallized by ethanol/water; yield 87%. TLC: toluene/ethyl acetate/acetic acid 8:2:2 *v*/*v*/*v*; ^1^H-NMR (CDCl_3_) δ 9.10 (s, 1H, H-5); 8.51 (s, 1H, H-2); 7.91 (d, 1H, H-6, *J* = 8.8 Hz); 7.88 (d, 1H, H-9, *J* = 2.4 Hz); 7.18 (dd, 1H, H-7, *J* = 8.8 Hz, *J* = 2.4 Hz); 7.01 (dd, 1H, H-3 pyrrole, *J* = 4.0 Hz, *J* = 1.3 Hz); 7.16 (m, 1H, H-5 pyrrole); 6.39 (dd. 1H, H-4 pyrrole, *J* = 4.0 Hz, *J* = 2.8 Hz); 4.08 (s, 3H, NCH_3_); 4.06 (s, 3H, OCH_3_). ^13^C-NMR (CDCl_3_) δ 179.2, 165.9, 155.4, 145.5, 130.0, 128.9, 120.8, 117.3, 107.5, 106.4, 55.8, 37.8; ESI-MS calcd for C_17_H_14_N_4_O_2_ (306.32); found: *m*/*z* 307.02[M + H]^+^. Anal. C_17_H_14_N_4_O_2_ (C, H, N).

### 3.10. 8-Methoxypyrazolo[1,5-a]quinazolin-5(4H)-one (***7***)

The decarboxylation of the ethyl 8-methoxy-5-oxo-4,5-dihydropyrazolo[1,5-a]quinazoline 3-carboxylate [18] (1.0 mmol) was obtained in H_3_PO_4_ at fusion condition. After that starting material disappeared in TLC (toluene/ethyl acetate/methanol 8:2:1.5 *v*/*v*/*v* as eluent), the treatment with ice/water gave a precipitate that was isolated and purified by recrystallization with ethanol. Cream crystals, yield 80%; IR ν cm^−1^ 3137, 1697; ^1^H-NMR (DMSO-d_6_) δ 12.05 (bs, 1H, NH, exch.); 8.02 (d, 1H, H-6, *J* = 8.8 Hz); 7.76 (d, 1H, H-2, *J* = 2.0 Hz); 7.47 (d, H-9, *J* = 2.4 Hz); 7.02 (dd, 1H, H-7, *J* = 8.8 Hz, *J* = 2.4 Hz); 5.87 (d, 1H, H-3, *J* = 2.0 Hz); 3.92 (s, 3H, OCH_3_). ESI-MS calcd for C_11_H_9_N_3_O_2_ (215.21); found: *m*/*z* 215.91[M + H]^+^. Anal. C_11_H_9_N_3_O_2_ (C, H, N).

### 3.11. 8-Methoxypyrazolo[1,5-a]quinazoline (***8***)

From **7**, following the same procedure described for the synthesis of **5a**–**e**. White crystals by ethanol; yield 85%. ^1^H-NMR (DMSO-d_6_): δ 8.95 (s, 1H, H-5); 8.16 (d, 1H, H-2, *J* = 2.2 Hz), 8.11 (d, 1H, H-6, *J* = 8.8 Hz); 7.75 (d, 1H, H-9, *J* = 2.4 Hz); 7.22 (dd, 1H, H-7, *J* = 8.8 Hz, *J* = 2.4 Hz); 6.77 (d, 1H, H-3, *J* = 2.2 Hz); 3.99 (s, 3H, OCH_3_). ESI-MS calcd for C_11_H_9_N_3_O (199,21); found: *m*/*z* 199.81[M + H]^+^. Anal. C_11_H_9_N_3_O (C, H, N).

### 3.12. General procedure for the synthesis of ***9a**,**b***

A solution of starting material **8** (0.35 mmol) in dichloromethane (5 mL) was supplemented with an excess of bromine (0.8 mL) to obtain compound **9a** or *N*-iodosucinimide (NIS, 1:2) for final product **9b**. The final solution was evaporated to dryness, and the residue was recovered with 10% NaOH solution, filtered, and washed with water. The raw compound was recrystallized by a suitable solvent.

#### 3.12.1. 3-Bromo-8-methoxypyrazolo[1,5-a]quinazoline (**9a**)

From **5e** and bromine; yield 85%; ^1^H-NMR (DMSO-d_6_): δ 9.03 (s, 1H, H-5); 8.33 (1, 1H, H-2), 8.16 (d, 1H, H-6, *J* = 8.8 Hz); 7.73 (d, 1H, H-9, *J* = 2.4 Hz); 7.26 (dd, 1H, H-7, *J* = 8.8 Hz, *J* = 2.4 Hz); 4.00 (s, 3H, OCH_3_). ^13^C-NMR (DMSO-d_6_) δ 155.4, 133.6, 128.9, 120.8, 106.4, 91.9, 55.8. ESI-MS calcd for C_11_H_8_N_3_OBr (278.10) found: *m*/*z* 278.7 [M + H]^+^. Anal. C_11_H_8_N_3_OBr (C, H, N). Anal C, H, N.

#### 3.12.2. 3-Iodo-8-methoxypyrazolo[1,5-a]quinazoline (**9b**)

From **5e** and NIS; white crystals; yield 85%. ^1^H-NMR (DMSO-d_6_): δ 9.09 (s, 1H, H-5); 8.27 (s, 1H, H-2), 8.15 (d, 1H, H-6, *J* = 8.8 Hz); 7.73 (d, 1H, H-9, *J* = 2.2 Hz); 7.25 (dd, 1H, H-7, *J* = 8.8 Hz, *J* = 2.2 Hz); 4.00 (s, 3H, OCH_3_). ^13^C-NMR (DMSO-d_6_) δ 155.4, 138.1, 128.9, 120.8, 117.3, 106.4, 56.3, 55.8. ESI-MS calcd for C_11_H_8_N_3_OI (325.11); found: *m*/*z* 325.71[M + H]^+^. Anal. C_11_H_8_N_3_OI (C, H, N).

### 3.13. General procedure for the synthesis of ***11a**,**b**,**d**–**g***

NaBH_3_CN (0.5 mmol) was added to a solution of starting material, **6a**,**b**,**d**–**g** (0.15 mmol), in glacial acetic acid (5 mL); the mixture was maintained at 50 °C for 1 h, and the reaction was monitored by TLC (toluene/ethyl acetate/methanol 8:2:1.5 *v*/*v*/*v* as eluent). When the starting material disappeared, the mixture was cooled and water was added; the precipitate was collected by filtration and purified by recrystallization.

#### 3.13.1. 3-(2-Methoxyphenylcarbonyl)-8-methoxy-4,5-dihydropyrazolo[1,5-a]quinazoline (**11a**)

From **6a**, pale cream crystals, yield 68%; IR ν cm^−1^ 3404, 1639; ^1^H-NMR (DMSO-d_6_) δ 7.82 (bs, 1H, NH, exch.); 7.44 (t, 1H, H-4 phenyl, *J* = 8.4 Hz); 7.32 (s, 1H, H-2); 7.27 (d, 1H, H-6 phenyl, *J* = 7.6 Hz); 7.20 (d, 1H, H-6, *J* = 8.4 Hz); 7.12 (d, 1H, H-3 phenyl, *J* = 8.4 Hz); 7.05 (d, H-9, *J* = 2.0 Hz); 7.04 (t, 1H, H-5 phenyl, *J* = 7.6 Hz); 6.74 (dd, 1H, H-7, *J* = 8.4 Hz, *J* = 2.0 Hz); 4.52 (s, 2H, CH_2_); 3.75 (s, 3H, 8-OCH_3_); 3.74 (s, 3H, OCH_3_). ^13^C-NMR (DMSO-d_6_) δ 196.3, 160.1, 149.6, 141.7, 134.7, 133.6, 130.9, 127.3, 126.6, 123.9, 117.4, 114.4, 112.2, 111.7, 104.6, 99.6, 55.6, 42.4. ESI-MS calcd for C_19_H_17_N_3_O_3_ (335,36); found: *m*/*z* 335.96[M + H]^+^. Anal. C_19_H_17_N_3_O_3_ (C, H, N).

#### 3.13.2. 3-(Thien-2-ylcarbonyl)-8-methoxy-4,5-dihydropyrazolo[1,5-a]quinazoline (**11b**)

From **6b**, pale yellow crystals, yield 74%; IR ν cm^−1^ 3430, 1640; ^1^H-NMR (DMSO-d_6_) δ 8.19 (s, 1H, H-2); 7.96 (d, 1H, H-3 thienyl, *J* = 3.2 Hz); 7.91 (d, 1H, H-5 thienyl, *J* = 4.4 Hz); 7.84 (bs, 1H, NH, exch.); 7.23 (m 2H, H-6 and H-4 thienyl); 7.10 (d, 1H, H-9 *J* = 2.0 Hz); 6.76 (d, 1H, H-7, *J* = 8.4); 4.53 (s, 2H, CH_2_); 3.77 (s, 3H, OCH_3_). ^13^C-NMR (DMSO-d_6_) δ 177.9, 159.7, 150.4, 144.5, 141.3, 134.2, 132.0, 131.3, 129.6, 128.2, 112.6, 111.9, 103.3, 99.8, 55.9, 42.2.. ESI-MS calcd for C_16_H_13_N_3_O_2_S (311,36); found: *m*/*z* 312.06[M + H]^+^. Anal. C_16_H_13_N_3_O_2_S (C, H, N).

#### 3.13.3. 3-(4-Methoxyphenylcarbonyl)-8-methoxy-4,5-dihydropyrazolo[1,5-a]quinazoline (**11d**)

From **6d**, pale cream crystals, yield 50%; IR ν cm^−1^ 3317, 1633; ^1^H-NMR (DMSO-d_6_) δ 7.82 (m, 2H, H-2 and NH exch.); 7.77 (d, 2H, H-2, and H-6 phenyl, *J* = 8.0 Hz); 7.21 (d, 1H, H-6, *J* = 8.4 Hz); 7.10 (s, 1H, H-9); 7.04 (d, 2H, H-3, and H-5 phenyl, *J* = 8.0 Hz); 6.75 (d, 1H, H-7, *J* = 6.8 Hz); 4.53 (s, 2H, CH_2_); 3.82 (s, 3H, 8-OCH_3_); 3.76 (s, 3H, OCH_3_). ^13^C-NMR (DMSO-d_6_) δ 187.1, 165.3, 163.2, 154.5, 145.9, 142.6, 137.7, 130.5, 128.4, 117.3, 114.3, 113.8, 111.7, 99.67, 56.8, 55.9, 42.0. ESI-MS calcd for C_19_H_17_N_3_O_3_ (335,36); found: *m*/*z* 334.76[M + H]^+^. Anal. C_19_H_17_N_3_O_3_ (C, H, N).

#### 3.13.4. 3-(Fur-2-ylcarbonyl)-8-methoxy-4,5-dihydropyrazolo[1,5-a]quinazoline (**11e**)

From **6e**, pale yellow crystals, yield 80%; IR ν cm^−1^ 3325, 1680; ^1^H-NMR (CDCl_3_) δ 8.25 (s, 1H, H-2); 7.63 (s, 1H, H-5 furyl); 7.27 (m, 2H, H-9 and H-3 furyl); 7.17 (bs, 1H, NH exch.); 7.02 (d, 1H, H-6, *J* = 8.4 Hz); 6.70 (dd, 1H, H-7, *J* = 8.8 Hz, *J* = 2.0 Hz); 6.57 (m, 1H, H-4 furyl); 4.67 (s, 2H, CH_2_); 3.85 (s, 3H, OCH_3_). ^13^C-NMR (CDCl_3_) δ 142.0, 130.4, 127.4, 117.8, 116.0, 112.2, 112.1, 99.7, 55.6, 42.4. ESI-MS calcd for C_16_H_13_N_3_O_3_ (295.29); found: *m*/*z* 295.99[M + H]^+^. Anal. C_16_H_13_N_3_O_3_ (C, H, N).

#### 3.13.5. 3-(1H-pyrrol-2-ylcarbonyl)-8-methoxy-4,5-dihydropyrazolo[1,5-a]quinazoline (**11f**)

From **6f**; yield 25%; IR ν cm^−1^ 3354, 1682; ^1^H-NMR (CDCl_3_) δ 9.54 (bs, 1H, NH pyrrole, exch.); 8.01 (s, 1H, H-2); 7.29 (d, 1H, H-9, *J* = 2.0 Hz); 7.03 (m, 4H, H-6, NH exch., H-3 and H-5 pyrrole); 6.70 (dd, 1H, H-7, *J* = 8.8 Hz, *J* = 2.0 Hz); 6.36 (m. 1H, H-4 pyrrole); 4.65 (s, 2H, CH_2_); 3.86 (s, 3H, OCH_3_). ^13^C-NMR (CDCl_3_) δ 178.8, 160.1, 149.6 141.7, 134.7, 130.9, 129.6, 127.3, 117.4, 112.2, 111.7, 108.0, 104.6, 99.6, 55.6, 42.4. ESI-MS calcd for C_16_H_14_N_4_O_2_ (294.31); found: *m*/*z* 293.61[M + H]^+^. Anal. C_16_H_14_N_4_O_2_ (C, H, N).

#### 3.13.6. 3-(1-Methyl-1H-pyrrol-2-ylcarbonyl)-8-methoxypyrazolo[1,5-a]quinazoline (**11g**)

From **6g**; yield 70%. ^1^H-NMR (CDCl_3_) δ 7.85 (s, 1H, H-2); 7.27 (d, 1H, H-9, *J* = 2.4 Hz); 7.00 (m, 2H, H-6 and NH exch.); 6.91 (m, 1H, H-3 pyrrole); 7.16 (m, 1H, H-5 pyrrole); 6.69 (d, 1H, H-7, *J* = 8.8); 6.17 (m. 1H, H-4 pyrrole); 4.62 (s, 2H, CH_2_); 3.93 (s, 3H, NCH_3_); 3.85 (s, 3H, OCH_3_). ^13^C-NMR (CDCl_3_) δ 178.8, 160.1, 149.6, 141.7, 134.7, 130.9, 129.5, 127,3, 117.3, 112.2, 111.7, 108.0, 104.6, 99.6, 55.6, 42.4, 36.8; ESI-MS calcd for C_17_H_16_N_4_O_2_ (308.33); found: *m*/*z* 308.93[M + H]^+^. Anal. C_17_H_16_N_4_O_2_ (C, H, N).

### 3.14. Radioligand Binding Assay

[^3^H]Ro15-1788 (specific activity 78.8 Ci/mmol) was obtained from Perkin Elmer. All of the other chemicals, which were of reagent grade, were obtained from commercial suppliers. Bovine cerebral cortex membranes were prepared as previously described [28,29]. The membrane preparations were diluted with 50 mM of tris-citrate buffer pH 7.4, and used in the binding assay. Protein concentration was assayed using the method of Lowry et al. [30]. [^3^H]Ro 15-1788 binding studies were performed as previously reported [21]. At least six different concentrations of each compound were used. The data of *n* = 5 experiments carried out in triplicate were analyzed by means of an iterative curve-fitting procedure (program Prism, GraphPad, San Diego, CA), which provided IC_50_, Ki, and SEM values for tested compounds, the Ki values being calculated from the Cheng and Prusoff equation [31].

### 3.15. General Methods for Electrophysiological Assays

#### 3.15.1. Expression of Human Receptor Subunits

A mixture of pCDM8-based vectors for the α_1_, β_2_, or γ_2L_ subunits of human GABA_A_ receptors (total of 1.5 ng of DNA, comprising equal amounts of α, β, and γ subunit vectors), or equal amounts of α and β receptors for the expression of α_1_β_2_ receptors, were injected into the animal pole of *X. laevis* oocytes as described [32] with the use of a microdispenser (Drummond Scientific, Broomwall, PA). The injected oocytes were maintained at 13 °C in sterile modified Barth’s solution [MBS: 88 mM NaCl, 1 mM KCl, 10 mM HEPES-NaOH (pH 7.5), 0.82 mM MgSO_4_, 2.4 mM NaHCO_3_, 0.91 mM CaCl_2_, 0.33 mM Ca(NO_3_)_2_] supplemented with streptomycin (10 mg/L), penicillin (10,000 U/L), gentamicin (50 mg/L), theophylline (90 mg/L), and pyruvate (220 mg/L).

#### 3.15.2. Electrophysiology

Electrophysiological measurements were performed in oocytes 2 to 4 days after DNA injection. Oocytes were placed in a rectangular chamber (volume ~100 µL) and perfused at a rate of 1.7 mL/min with MBS at room temperature with the use of a roller pump (Cole-Parmer, Chicago, IL) and 18-gauge polyethylene tubing (Clay Adams, Parsippany, NJ, USA). Oocytes were impaled at the animal pole with two glass electrodes (0.5 to 10 MΩ) filled with 3 M of KCl, and were clamped at −70 mV with the use of an oocyte clamp (model OC725C; Warner Instruments, Hamden, CT). Currents were measured and analyzed with the pClamp 9.2 software (Molecular Devices, Union City, CA, USA). GABA (Sigma, St. Louis, MO, USA) was dissolved in MBS and applied to the oocytes for 30 s. Oocytes were perfused with test drugs for 30 s either in the absence of the agonists or in its presence at the EC_5–10_ (the concentration of agonist that induces a peak current equal to 5–10% of the maximal current elicited by the maximal concentration of the agonist). Compounds were first dissolved in DMSO at a concentration of 10 mM, and then diluted in MBS to the final concentrations. In each experiment, control responses were determined before and 10/15 min after application of the drug.

#### 3.15.3. Statistics

Statistical analysis was performed on normalized data using the Kruskal–Wallis test followed by Dunn’s post hoc test or the Mann–Whitney test using GraphPad Prism 7 (Graph Pad Software, Inc., San Diego, CA, USA).

## 4. Conclusions

In this paper, new 8-methoxypyrazolo[1,5-a]quinazolines bearing at position 3 of the (hetero)aryl group (type **5** compounds) or (hetero)aroyl moiety (type **6** compounds) and their corresponding 4,5-dihydroderivatives (type **11** compounds) were synthesized and evaluated for their ability to modulate the recombinant α_1_β_2_γ_2L_ GABA_A_ receptors. Compounds that showed a certain modulation of chlorine current are 3-(hetero)aroylpyrazolo[1,5-a]quinazolines, and **6a** and **6b** were the most representative compounds. These products modulate the GABA_A_R in an opposite manner, suggesting that **6b** acts as partial agonist and **6a** acts as an inverse partial agonist.

Among the 3-(hetero)aroyl derivatives with the 4,5-dihydropyrazolo[1,5-a]quinazoline scaffold, the most interesting compound was **11d**, for which an effect on the chlorine current is measurable at ≥0.01 μM. This compound will be the object of further studies.

Finally, we found the profile of the null modulator **6g** interesting, since it not only acts as an antagonist blocking the potentiation of the GABAergic function induced by diazepam (so interacting at the high-affinity benzodiazepine site located in the extracellular domain at the α+/β− [2]), but it also works as a positive allosteric modulator at the low-affinity site located in the extracellular domain at the α+/β− interface. These results suggest that this compound could act to both the high-affinity and the low-affinity benzodiazepine site, so we can hypothesize that it will display a much broader action than the classic high-affinity benzodiazepine site ligands.

Moreover, since **6g** is the first compound acting via the α+/β− interface of GABA_A_ receptors with the PQ scaffold, it may represent an interesting lead for the discovery of a new class of ligand, which would be useful for the study of this recently discovered low-affinity benzodiazepine site.

## Supplementary Materials

Supplementary materials can be found at https://www.mdpi.com/1422-0067/20/6/1438/s1.

## Abbreviations

GABA_A_R	GABA receptors Type A
GABA_B_R	GABA receptor Type B
GPCR	G-protein-coupled receptors
LGIC	ligand gated ion channel
PQ	pyrazoloquinazoline
PBTs	pyrazolobenzotriazines
PPs	pyrazolopyrimidines
PTs	pyrazolotriazines
