# Supplementary material for: Synthesis of New GABAA Receptor Modulator with Pyrazolo[1,5-a]quinazoline (PQ) Scaffold
Source: Int J Mol Sci. 2019 Mar 21;20(6):1438. doi: 10.3390/ijms20061438 (PMC6470557; doi:10.3390/ijms20061438)
Supplement: Supplementary file 1 [file ijms-20-01438-s001.pdf]

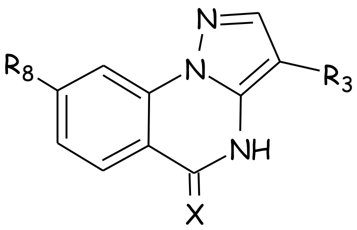

3a-h, 7, 11a, b, d-g

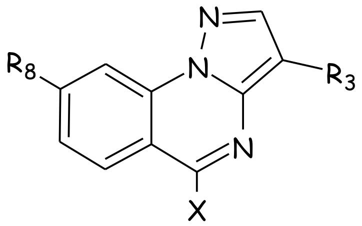

4a-c, 5a-i, 6a-g, 8, 9a, b, 10

Table S1: Chemical data for Pyrazolo[1,5-a]quinazoline derivatives

Comp.	R_3	R_8	X	Formula (MW)	mp °C<br>recryst. solvent
**3a**	phenyl	H	O		[1]
**3b**	2-thienyl	H	O	$C_{14}H_9N_3OS$ (267.31)	243-245 °C methoxyethanol
**3c**	3-thienyl	H	O	$C_{14}H_9N_3OS$ (267.31)	232-234 °C methoxyethanol
**3d**	2-thienyl	OMe	O	$C_{15}H_{11}N_3O_2S$ (297.33)	288-289 °C methoxyethanol
**3e**	3-thienyl	OMe	O	$C_{15}H_{11}N_3O_2S$ (297.33)	>300 °C ethanol
**3f**	CO-(2-OMe)-Ph	OMe	O	$C_{19}H_{15}N_3O_4$ (349,34)	122-125 °C ethanol
**3g**	CO-2-thienyl	OMe	O	$C_{16}H_{11}N_3O_3S$ (325,34)	204-205 °C ethanol
**3h**	CO-3-thienyl	OMe	O	$C_{16}H_{11}N_3O_3S$ (325,34)	207-208 °C ethanol
**4a**	CO-(2-OMe)-Ph	OMe	Cl	$C_{19}H_{14}N_3O_3Cl$ (367,79)	173-175 °C ethanol
**4b**	CO-2-thienyl	OMe	Cl	$C_{16}H_{10}N_3O_2SCl$ (343,79)	169-170 °C ethanol
**4c**	CO-3-thienyl	OMe	Cl	$C_{16}H_{10}N_3O_2SCl$ (343,79)	167-169 °C ethanol
**5a**	phenyl	H	H	$C_{16}H_{11}N_3$ (245,10)	148-150 °C ethanol 80%
**5b**	2-thienyl	H	H	$C_{14}H_9N_3S$ (251,31)	248-250 °C ethanol 80%
**5c**	3-thienyl	H	H	$C_{14}H_9N_3S$ (251,31)	253-255 °C ethanol 80%
**5d**	2-thienyl	OMe	H	$C_{15}H_{11}N_3OS$ (281,33)	158-160 °C ethanol 80%
**5e**	3-thienyl	OMe	H	$C_{15}H_{11}N_3OS$ (281,33)	161-163 °C ethanol 80%
**5f**	2-MeO-phenyl	OMe	H	$C_{18}H_{15}N_3O_2$ (305,33)	166-168 °C ethanol
**5g**	3-furyl	OMe	H	$C_{15}H_{11}N_3O_2$ (265,27)	158-160 °C ethanol
**5h**	2-(1-Boc-pyrrole)	OMe	H	$C_{20}H_{20}N_4O_3$ (364.40)	oil
**5i**	2-(1H)-pyrrole	OMe	H	$C_{15}H_{12}N_4O$ (264.28)	180-183 °C ethanol
**6a**	CO-(2-OMe)-Ph	OMe	H	$C_{19}H_{15}N_3O_3$ (333,34)	242-244 °C ethanol
**6b**	CO-2-thienyl	OMe	H	$C_{16}H_{11}N_3O_2S$ (309,34)	158-160 °C ethanol
**6c**	CO-3-thienyl	OMe	H	$C_{16}H_{11}N_3O_2S$ (309,34)	155-157 °C ethanol

**6d**	CO-(4-OMe)-Ph	OMe	H	$C_{19}H_{15}N_3O_3$ (333,34)	153-155 °C ethanol
**6e**	CO-2-furyl	OMe	H	$C_{16}H_{11}N_3O_3$ (293,28)	155-157 °C ethanol
**6f**	CO-2-(1H)-pyrrole	OMe	H	$C_{16}H_{12}N_4O_2$ (292,29)	220-222 °C ethanol/water
**6g**	CO-2-(1-methyl-1H-pyrrole)	OMe	H	$C_{17}H_{14}N_4O_2$ (306,32)	178-180 °C ethanol/water
**7**	H	OMe	O	$C_{11}H_9N_3O_2$ (215.21)	279-280 °C methoxyethanol
**8**	H	OMe	H	$C_{11}H_9N_3O$ (199,21)	139-141 °C ethanol
**9a**	Br	OMe	H	$C_{11}H_8N_3OBr$ (278.10)	196-197 °C ethanol 80%
**9b**	I	OMe	H	$C_{11}H_8N_3OI$ (325.11)	207-209 °C ethanol 80%
**10**	COOH	OMe	H		[2]
**11a**	CO-(2-OMe)-Ph	OMe	H, H	$C_{19}H_{17}N_3O_3$ (335,36)	220-222 °C ethanol
**11b**	CO-2-thienyl	OMe	H, H	$C_{16}H_{13}N_3O_2S$ (311,36)	177-178 °C ethanol
**11d**	CO-(4-OMe)-Ph	OMe	H, H	$C_{19}H_{17}N_3O_3$ (335,36)	141-143 °C ethanol
**11e**	CO-2-furyl	OMe	H, H	$C_{16}H_{13}N_3O_3$ (295,29)	148-151 °C ethanol
**11f**	CO-2-(1H)-pyrrole	OMe	H, H	$C_{16}H_{14}N_4O_2$ (294,31)	230-233 °C ethanol
**11g**	CO-2-(1-methyl-1H-pyrrole)	OMe	H, H	$C_{17}H_{16}N_4O_2$ (308,33)	240-242 °C ethanol

1. **Penning**, T. D.; Thomas, S. A.; Hajduk, P. J.; Sauer, D. R.; Sarris, K.; Giranda, V. L. Potent PARP Inhibitors WO2007149907 A2
2. **Guerrini**, G.; Ciciani, G.; Crocetti, L.; Daniele, S.; Ghelardini, C.; Giovannoni, M. P.; Iacovone, A.; Di Cesare Mannelli, L.; Martini, C.; Vergelli, C. Identification of a New Pyrazolo[1,5-a]quinazoline Ligand Highly Affine to γ-Aminobutyric Type A (GABAA) Receptor Subtype with Anxiolytic-Like and Antihyperalgesic Activity. *J. Med. Chem.* **2017**, *60*, 9691–9702, doi:10.1021/acs.jmedchem.7b01151.
